# Psychological Benefits and Challenges of Ph.D. Entrance Exam Virtual Interviews During COVID-19 Pandemic: Does Gender Play a Role?

**DOI:** 10.3389/fpsyg.2021.800715

**Published:** 2021-11-25

**Authors:** Saman Ebadi, Saba Bashiri

**Affiliations:** Faculty of Humanities, Razi University, Kermanshah, Iran

**Keywords:** virtual interviews, Ph.D. applicants, entrance interview, COVID-19, psycholog, challenge, gender

## Abstract

This study aimed to investigate the reflections of Iranian students on Ph.D. entrance exam interviews held virtually nationwide during the Pandemic. Two hundred thirty-one Ph.D. applicants were invited to fill out an anonymous online survey designed in Google Forms, 36 out of whom volunteered to participate in follow-up semi-structured interviews. Two ANOVA measures were used to investigate the possible influences of gender and prior virtual interview experience on the applicants’ perceptions. Thematic analysis was also adopted to explore the participants’ attitudes and perceptions toward virtual interviews (VI). Quantitative findings showed that Iranian Ph.D. applicants perceived VI as a flexible and helpful procedure that provided them with satisfactory interpersonal treatment. Still, they did not favor the procedure’s perceived behavioral control and ability to communicate information to perform procedural justice. Moreover, neither their gender nor their prior experience of the virtual interview was a predictor of their perspectives’ discrepancies. The thematic analysis of the qualitative data revealed that despite having some cognitive, personal, and financial benefits, VI posed some technical, communicative, and personal challenges. This study provides implications for universities and applicants who will use VI for different purposes in higher education.

## Introduction

Based on higher education standards, the nationwide written exam (called Ph.D. Konkur) is annually administered as the main criterion to enter Ph.D. programs in all fields of study in Iran. As a part of the recruitment process, the summoned applicants had to recourse to the particular city(s) and university(s) for which they were qualified based on the norm-referenced written exam to participate in on-site interviews. Interviews generally provide the universities with multiple lines of information on the applicants’ previous research experience and their interests to conduct future studies. They allow both applicants and the Ph.D. programs to get the most information and find the most qualified applicants (Al Saiegh et al., 2020). As a consequence of the COVID-19 pandemic, recruitment processes have undergone novel changes (Davis et al., 2021; Kenwell et al., 2021). Strielkowski (2020) refers to this situation as a digital revolution in academic and higher education centers and believes it to be only one of the consequences of the COVID-19 pandemic. Transiting to web-based platforms for holding interviews (Robinson et al., 2020) in response to the pandemic was the most efficient decision made by most Iranian universities to recruit Ph.D. applicants for the academic year 2020-2021. Clary (2021) states that holding such interviews *via* virtual environments instead of traditional face-to-face interviews presents a double-edged sword. But Strielkowski (2020) believes that online higher education has almost the same attributes as real life in all its aspects, such as defenses, entrance, and final exams.

The same changes in interview processes for recruitment purposes occurred in other educational sects, especially medical education, which was highly impacted by the pandemic (Bhardwaj et al., 2021) including recruiting medical students for residency (Davis et al., 2020; Foong, 2020; Shreffler et al., 2021), surgical training (Day et al., 2020; McKinley et al., 2020), and emergence medicine (Deiorio et al., 2019). The United States health care is dramatically impacted by the pandemic (Patel et al., 2020). All on-site interviews for residency applicants were suggested to be transmitted to virtual assessments to decrease the disease outbreak (Al Saiegh et al., 2020), posing different challenges for both programs and applicants (Sarac et al., 2020). The same challenges can also be taken advantage of as opportunities for systemic improvements (Hammoud et al., 2020). Previous literature shows that recruitment processes undertaken *via* virtual formats are cost and time-saving, more efficient than traditional ones, and allow an acceptable amount of self-representation (Chandler et al., 2019; Robinson et al., 2020; Tseng, 2020; Vining et al., 2020).

Hill et al. (2021b) reported the results of a survey study in which 30 program directors and 64 candidates took part to disclose their perceptions on virtual interviews (VI) for the Complex General Surgical Oncology (CGSO) fellowship. The overall results showed that both program directors and applicants perceived the VIs to be convenient as it was easy to use and posed no technical challenges. A surprising insight was that most applicants preferred traditional on-site interviews and valued them more. Davis et al. (2021) addressed the financial and programming issues of implementing VI for Otolaryngology Residency recruitment. Twelve single 15-min VIs were conducted *via* zoom for 12 applicants who took part in the following online survey to share their experiences and attitudes with the researchers. Although VIs proved to be more cost-efficient than traditional ones, the results also noted most applicants’ dissatisfaction with the platform’s visual quality, poor eye contact opportunities, and unexpected interruptions.

Previous research highlights the probable effects of gender and prior interview experience on interview procedures and outcomes (Huffcutt et al., 2011; Robles et al., 2013; Pogrebtsova et al., 2020). For instance, interviewees will tend to speak in more depth and length when the interviewer is of the same gender and vice versa (Alhojailan, 2020), which is a manifestation of the similarity-attraction paradigm (Byrne, 1961) more tendency toward similarity. Furthermore, gender differences may result in significantly different performance in interviews (Barbour and Sandy, 2014; Leduc et al., 2017) and different emotional experiences (Hannon, 2012), especially when involved in a computer-based evaluation (Harley et al., 2020). Concerning Ph.D. programs, Spronken-Smith et al. (2018) stated that gender is one of the contributing factors to Ph.D. completion rates in New Zealand. Likewise, having interview experience is an antecedent of interviewees’ performance. It can lead to more self-efficacy (Huffcutt et al., 2011) and more preparation to cope with technical challenges (McColl and Michelotti, 2019). We assume that gender and prior experience also moderate the applicants’ reactions to VI.

As undertaking online interviews have not been a routine part of the Ph.D. entrance process and is considered “uncharted waters” (Patel et al., 2020), no single study has been undertaken on the subject. In other words, to the researchers’ knowledge, research on the subject of online interviews has been mostly restricted to gaining insights on medical education applications’ reflections and a comparison of face-to-face and online interviews implementing different theoretical frameworks and approaches. The specific theoretical framework implemented in this study is Potosky’s (2008) framework of media attributes embedded in communication theory (Arthur et al., 2018). Potosky defines assessment administration media in terms of four attributes that may alter applicants’ reactions to the assessment process. These attributes are transparency, social bandwidth, interactivity, and surveillance. A media is transparent when it paves the way for an unblocked communication exchange where the individuals can ask or respond to questions in the way they intend to and desire. Social bandwidth refers to the extent of relevant information which a media allows to be presented during a communication exchange. The extent to which a media allows for mutual communication exchange and interaction for both communication parties is defined as media interactivity attribute. Finally, surveillance has to do with privacy and security concerns, i.e., the extent to which the person feels secure that the communication process is not controlled or intercepted by a third party. The aim of this research project has therefore been to tap on Iranian Ph.D. applicants’ reflections on the virtual interview process for Ph.D. entrance in light of Potosky’s framework, as the insights gained from these reflections can be helpful for those who will use these platforms (Hill et al., 2021b). Furthermore, there is still little information about its pros and cons and some technical guidelines for conducting them (Hill et al., 2021a). Besides, the possible effects of gender and prior experience with VI will be explored as individual differences have a moderating effect on applicants’ reactions and behaviors (Lukacik et al., 2020). More specifically, this study seeks to answer the following questions:

(1) What are the Iranian Ph.D. applicants’ reflections on online entry interviews in terms of its perceived benefits and associated challenges?
(2) Do the gender and prior online interview experience affect their reflections?

## Literature Review

### Media Attributes Framework

One of the recent frameworks implemented in technology-mediated interview research was proposed by Denise Potosky (2008). She offered this conceptual framework to rethink candidates/personnel assessment “as a communication exchange process” and providing “new insight into the role of the medium used to administer tests, interviews, and surveys” (p. 629). Inspired by Barry and Fulmer (2004)’s study on social influence interactions in organizations using different media channels, Potosky distinguished among various media in terms of four distinct attributes: transparency, social bandwidth, interactivity, and surveillance. Unobtrusive and transparent media facilitate the communication process, while obtrusive media let the parties notice the mediating role of technology in the process. Social bandwidth is defined as “the maximum transfer rate of a medium” (p. 636), which may be limited in VI as they do not allow a complete representation of both interviewees and interviewers (Toldi, 2011). As its name suggests, the interactivity of a medium refers to the rate of exchange quality and feedback pace it provides. Surveillance refers to “publicness of interaction” (Barry and Fulmer, 2004, p. 275) and the level of information and communication security that the media presents.

From its inception as a viable framework for technological media research, the media attributes framework has often been used as the main or subsidiary framework to compare different media for selection and assessment purposes. Basch et al. (2020a) surveyed 154 working individuals to investigate their perceptions on three different interview types, i.e., face-to-face, synchronous (videoconferencing), and asynchronous. The participants perceived technology-based interviews, primarily when held asynchronously, as unfair compared to face-to-face traditional interviews since they could not present themselves fully due to physical absence, thus suffering from impaired social presence. In another investigation aiming to identify factors contributing to different perceptions and performance ratings in traditional interviews and VI, Basch et al. (2020b) surveyed 114 students *via* pre- and post-interview questionnaires. As expected by the researchers, VI was rated lower than a traditional interview. They perceived the VI as inadequate in providing social presence, eye contact, and impression management opportunities. In the same vein, comparing conventional and technology-mediated selection interviews, Melchers et al. (2021) concluded that different interview media bring about different performance ratings and perceptions under their inherent attributes.

### Virtual Interviews During Pandemic

Although the COVID-19 disease preceded the advent of virtual interviewing as a norm for Ph.D. applicant selection in Iran, online platforms have previously been utilized for the same purposes, especially for business hiring purposes (Joshi et al., 2020), even when there were no regulations for the required social distancing. It has been argued that as these regulations will remain, VIs will probably be utilized as a routine for selection purposes for the near future (McKinley et al., 2020; Hill et al., 2021a). Different platforms have been utilized in this regard, each of which has contributed to both applicants and program executers’ benefits and challenges. Many research projects have been carried out in 2020 and 2021 concerning the COVID-19 era, some of which are reported here.

Advanced endoscopy fellowship applicants and program directors were surveyed by Kamboj et al. (2021) to explore their experiences with VIs for the 2020 application cycle during the COVID-19. Research Electronic Data Capture was used to collect data which included 17-question surveys sent individually to the respondents. The survey was responded to by 37 applicants and 71 interviewers. Most applicants and interviewers were pleased with experiencing VIs. Specifically, the interviewers reported a good understanding of applicants’ background, interpersonal skills, professionalism, and career aspirations. On the other hand, thirty-four of them were doubtful about their ability to feature the endoscopy units and facilities *via* the platform. The applicants admitted that this experience was above their expectations as they understood the clinical responsibilities, the academic and educational expectations, procedural volume, and job placement at the program. The major limitation associated with VIs for them was that they did not get a good feel for the program and institution or the endoscopy unit.

A university-affiliated children’s hospital was the site for a study by Lewit and Gosain (2021) who distributed surveys among applicants and faculty who had experienced VIs in the Pediatric Surgery fellowship program. The surveys explored overall satisfaction with both on-site interviews and VIs, the quality of getting to know each other using each interview type, the impact of interview type of the final rank list, and whether they recommend VIs for future interview programs not. In contrast to the previous research findings, most faculty (75%) and applicants (87.5%) admitted on-site interviews over VIs. Consequently, they did not recommend in-person interviews to be replaced by VIs. Although the applicants believed that interview type impacted their final rank list, the faculty did not perceive such an influence. The applicants were displeased about getting to know the faculty and the program in this way. With all these aspects in mind, both applicants and faculty pointed to the potentiality of using VIs as a subsidiary to in-person ones, most likely as a screening tool.

Bamba et al. (2021) explored applicants’ opinions at the Indiana University Independent Plastic Surgery program to find out whether they prefer VI or in-person interviews and compare the perceptions of virtually interviewed applicants with in-person interviewed applicants. Online surveys were email distributed to 30 applicants, out of whom 18 responded to the survey (10 had completed the in-person interview and eight had completed VI). Findings indicated VI’s efficiency in both financial and temporal terms. But virtually interviewed applicants complained about lagging behind their counterparts as they did not get to know the program and the applicants in conventional interviews. For the same reason, all of them preferred the traditional interview type. On the other hand, the in-person group perceived their experience more positively and only 10% of them admitted to preferring VI.

## Materials and Methods

### Study Design

We performed a sequential explanatory mixed-methods study design (Creswell et al., 2003; Riazi, 2016), the QUAN-QUAL type (Dornyei, 2007), to collect quantitative data followed and substantiated by qualitative data. Therefore, the participants’ answers to an online survey were analyzed quantitatively and the data from individual asynchronous online interviews were analyzed *via* qualitative thematic analysis.

### Participants

All participants of this study (*N* = 231) were Ph.D. applicants who had experienced Ph.D. programs’ entrance online interviews in at least one Iranian university. The participants’ contact information was made available to the researchers after passing some administrative processes. Ethical considerations were ensured by explaining the study’s purposes and asking them to voluntarily fill out the corresponding online survey. Those who completed the survey were also requested to participate in the asynchronous interviews held *via* WhatsApp voice messaging contentedly. Nobody was obliged to take part in any stage and all of them were assured that their non-participation and responses to the survey and interview questions would not impact the results of the preceding Ph.D. interview. The demographic information of the participants is presented in Table 1.

**TABLE 1:** Demographics of applicants who completed the survey.

**Gender**	
-Male	111(49%)
-Female	120(51%)
**Age**	
-Range	24–46
**Major**	
-TEFL	129(56%)
-Linguistics	102(44%)

### Instruments

Quantitative data was collected online using Google Forms. The online survey consisted of three demographic questions (age, gender, and major), three general questions [any experience of taking part in an online interview, the primary method of gaining information about the interview staff (item 1), and the criterion for selecting the supervisor (item 2)], and 48 Likert-point statements in which respondents rated their level of agreement from strongly agree to strongly disagree. These statements were either self-developed or adopted from different sources (Chapman et al., 2003; Toldi, 2010; Langer et al., 2017; Basch and Melchers, 2019) and were arranged within thirteen subscales, including overall favorability (items 3–9), procedural justice/global fairness (items 10–13), ability to communicate information/opportunity to perform (items 14–18 adapted and items 19–23 self-developed), consistency (item 25), perceived flexibility/travel constraints (items 26–29), perceived ease of use (items 30–32), perceived usefulness (item 33), emotional creepiness (item 34), creepy ambiguity (items 35 and 36), privacy concerns (items 37–40), perceived behavioral control (items 41–47), two-way communication (items 48 and 49), and interpersonal treatment (item 50). Appendix A consists of the English version of this survey, although all the items were translated into Persian for this study purpose in order to avoid any miscomprehensions. The questionnaire was piloted *via* consulting an expert and researcher in the field and having four non-participants answer the questions; consequently, some items were removed and some others modified. The Cronbach’s alpha coefficient of the questionnaire was 0.83. We asked the respondents to take part in the asynchronous online interviews to provide us with more explanations for collecting the qualitative data. The semi-structured interview questions were directly extracted from the survey subscales (four questions/Appendix B).

### Procedure

The whole data collection procedure was conducted over 4 weeks. First, the researchers created a group in WhatsApp messenger, including 300 Ph.D. applicants, the contact information of whom was accessed legally from the university headquarters. The researchers provided the members with a general overview of the whole process as group admins, followed by the link that redirected them to the online survey. Those who did not acquiesce to either left the group or did not respond (*N* = 69). Three weeks after creating the group, the researchers asked the respondents to participate in the online interviews conducted *via* the end-to-end encrypted WhatsApp chats. A total of 36 applicants agreed to take part. The interviews were conducted in Persian to avoid any misunderstanding on the part of the interviewees. Each interview session lasted 7–10 min, all the audios were saved, translated into English, and transcribed verbatim.

### Data Analysis

A 26.0 SPSS statistics package was used to analyze the quantitative data. To address the first research question, descriptive statistics (percentage) was applied. Moreover, the qualitative data were analyzed using thematic analysis. All the audios were transcribed, translated into English, and read several times to be segmented into separate units or themes. Referential statistics (ANOVA) was utilized to address the second research question.

## Results

### Candidates’ Reflections on Perceived Benefits and Associated Challenges of Ph.D. Online Interviews

**FIGURE 1:**
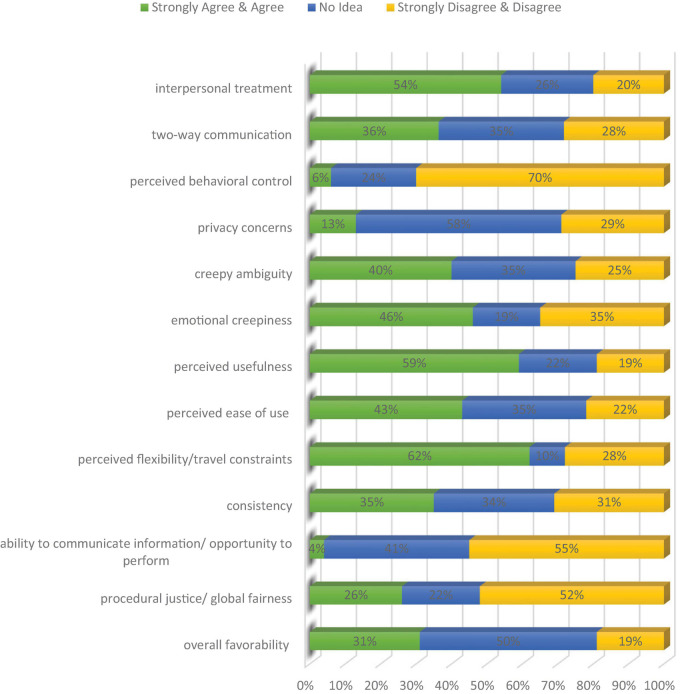
Analysis of participants’ survey responses.

Research question one concerned how Ph.D. applicants perceive the online interview as a Ph.D. student selection medium. The survey results, divided by its subscales, are summarized in Figure 1. The percentages of negative and positive options are summed up under a single category for saving space and being more reader-friendly.

Overall, most of the applicants were in favor of online interviewing. The most positively responded factor out of the 13 questionnaire subscales was perceived flexibility/travel constraints (62%). Likewise, 59% of them perceived the procedure as helpful, and 54% were satisfied with interpersonal treatment during the interview. On the other hand, they were significantly less satisfied with perceived behavioral control (4%) and the ability to communicate information/opportunity to perform (6%). In conjunction, 52% did not perceive this procedure as a proper selection tool. Furthermore, they were primarily unsure about privacy concerns and the overall favorability of the interview process.

The individual semi-structured online interviews were used to explore the applicants’ perceived benefits and associated challenges of Ph.D. online interviews. The thematic analysis of the interview data resulted in generating broad themes, as presented in Table 2. Each emergent theme is elaborated on by quoting applicants’ statements anonymously (A1–A36).

**TABLE 2:** Themes and codes extracted from the interview data.

A. Cognitive benefits	1. Less frustration
	2. More self-confidence
	3. More concentration on the questions
B. Financial benefits	1. No travel costs
	2. No residency costs
C. Personal benefits	1. More time to get prepared
	2. More safety
	3. Less fatigue
	4. More chance to take part in multiple interviews
	5. No need to be absent for job
D. Technical challenges	1. Webcam problems
	2. Speaker and microphone problems
	3. Connectivity problems
	4. Platform-related problems
E. Communicative challenges	1. Not enough feedback
	2. Less chance to self-represent
	3. No access to all professors’ video/audio
F. Personal challenges	1. Non-friendly atmosphere
	2. Not feeling an academic atmosphere
	3. Not suitable for all personality types
G. Ways to improve	1. Holding webinars
	2. Sharing tutorial videos

The cognitive benefits of VI were the opportunities they were provided as they felt less frustrated, more self-confident, more concentrated, and given more chances to represent themselves. A1, A23, and A4’s comments are as follows, respectively:

*Last year Ph.D. interviews were conducted in person. I do not forget how much I suffered from frustration in being in such a situation. I mean having to present myself in front of several experts of the field. Fortunately, this feeling was suppressed to a great degree as I was not in the same atmosphere.*

*You know, for being selected as a Ph.D. candidate, you have to convince the professors that you believe in your abilities. The key to bringing about such a belief in them is self-confidence. In my opinion, VI provided me with good self-confidence as I was not worried about miscellaneous issues.*

*The more you trust yourself, the more you will succeed in handling the situation at hand. As a result of more confidence, I was entirely concentrated on the posed questions and was more apt to take part in the discussions.*

Financial issues were the most favorable factors being mentioned by the participants. In this regard, A5 and A20 breathed a sigh of relief as their comments reveal:

*I have some bad memories of not traveling to the interview site because of financial issues. I was invited for an interview by several universities. Still, I could not afford to participate in all of them. The most important reason had to do with monetary issues, which were alleviated this year.*

*Besides and following financial issues, one of the most important reasons for not traveling to the invited universities was having no place to stay overnight. You know you need to rest and relax. No hotel or residence other than your place of habitant can be so relaxing.*

Factors other than cognitive and financial ones were more related to the applicants themselves and their families:

*If I had to travel to another city; especially far cities, I had to plan a different schedule because I missed 2 days, i.e., 1 day for traveling and 1 day for returning. This year, I had more time to study and get prepared for the interview day. (A28)*

*I am sure that if the interviews were in person, I could not convince my family to take part in most of them*… *due to both transportation and COVID issues. (A7)*

*Traveling to other cities causes a lot of fatigue in me, thus impacting my performance in the interview. I was no more worried about this issue. (A12)*

*Several universities invited me and if the interviews were not held virtually, I had to select among them based on some criteria. VI gave me more chance of being accepted as I could participate in interviews at multiple universities. (A19)*

*If the interviews had to be conducted on-site, I had to ignore participating in some of them. I am a full-time employe. I had to quit my job, but my absence from the job was limited, unlike last year that I had to recess it for at least 1 or 2 days. (A29)*

Technical challenges were the most cited factors that hindered applicants’ performance. The following comments are representatives of these challenges:

*During VI, I had some problems receiving and sending voice and video despite having a stable connection. This led to my frustration and anxiety. (A11)*

*Virtual platforms cannot be considered as the most convenient tool for conducting such essential interviews. I suffered from poor internet quality. As a result, I did not perform well despite my good preparation. (A2)*

*I was so bothered to download the platform and its supplementary apps. I wonder whether this was the case for others or not. Furthermore, I lost the page abruptly. (A10)*

Other challenging factors were primarily relevant to interactional and communicative issues characteristic of virtual environments.

*Interaction is a determining factor when negotiating an issue. I did not receive sensible feedback from the professors, maybe because I could not fully understand their facial expressions and body language. (A4)*

*Being interviewed means self-representation. It means showing your best side to get the interviewers’ attention. Based on my previous experience with in-person interviews, the chance to represent yourself in an online interview is not comparable to that in on-site interviews due to time limitations. I was undecided. (A25)*

*The interview team should work interactively, not individually. When you can see and hear all the interviewers, you feel more decided and less frustrated. This feature was absent in VI. I did not receive the required feedback. You know, when I see my partner eager to hear me, I can go on quickly. (A34)*

The last category had to do with challenges personally, as some may not feel so comfortable with technology-based interactions. The following statements point out these challenges:

*In-person interviews happen in a more friendly and personal atmosphere which contributes to applicants’ lower anxiety*… *meeting professors in person can totally alter the interviewees’ performance. Unfortunately, this was not the case with VI. (A18)*

*For me, an on-site interview seems more academic and brings about a sense of formality. I could not take VI as seriously because I thought it would be totally different from traditional interviews in terms of its atmosphere and ability to perform. (A31)*

*I am not so comfortable with performing via virtual platforms. I prefer actual presence and see and hear the interviewers in person. I do not believe in my virtual abilities; I fear being virtually interviewed even for reasons other than Ph.D. entrance (A10)*

Apart from the benefits and challenges associated with VI, the participants recommended holding webinars and sharing tutorial videos as two ways leading to a better VI experience. A3 and A6, respectively believed that:

*One of the innovative ways of discussing different aspects of an issue is holding online seminars, i.e., webinars, which allow participation everywhere and anytime. If such a webinar was held by the Ministry of Science, Research, and Technology or the university, we had more familiarity and would be more successful in the interview.*

*As platforms to hold online meetings abound, each specific university can share links to tutorial videos on running and using the needed platform on its website.*

### Gender and Prior Experience Influences

Addressing the second research question, two sets of ANOVA were calculated to investigate possible differences between gender and prior experience. As Tables 3, 4 show, neither gender nor the prior experience of taking part in an online interview impacted the applicants’ reflections on the procedure (sig > 0.05).

**TABLE 3:** ANOVA results for the influence of gender differences.

	**Sum of squares**	**Df**	**Mean square**	***F***	**Sig.**
Between groups	0.046	1	0.046	0.247	0.620
Within groups	43.045	229	0.188		
Total	43.091	230			
					

**TABLE 4:** ANOVA results for the influence of prior experience.

	**Sum of squares**	**df**	**Mean square**	***F***	**Sig.**
Between groups	0.625	1	0.625	3.372	0.068
Within groups	42.466	229	0.185		
Total	43.091	230			

The applicants’ responses to the survey questions showed their opinions about the interview process and the staff. Gender is one of the factors impacting both evaluating and making decisions about programs (Nesdoly et al., 2020), although it was not the case in this study. Likewise, having prior experience with VI makes a difference between applicants’ reflections. One possible reason for this non-discrepancy is due to the design differences of different VI platforms, such as the opportunity to fake responses (Lukacik et al., 2020) and restrictiveness in making and correcting errors (Wong, 2020). Another explanation is that experiencing the actual interview lowers the differences between the applicants with prior experience and their counterparts (Melchers et al., 2021). Unlike this study, Wong (2020) found that having no prior VI experiences results in more negative reactions against VI, especially concerning fairness and justice issues, but experiencing VI may help to mitigate pre-interview negative reflections (Basch et al., 2020a). According to Robles et al. (2013), adverse effects of having no VI experience can be counteracted by providing the applicants with pre-interview preparation materials.

## Discussion

The COVID-19 pandemic disrupted in-person Ph.D. entrance interviews across many fields. The recruitment/selection process is one of the beneficiaries of technological advancement (Nikolaou, 2021) and Ph.D. entrance interviews are not an exception, especially during the pandemic. This study evaluated perceptions regarding virtual Ph.D. entrance interviews. It demonstrated that the virtual interview process was an efficient process for applicants regarding perceived flexibility, usefulness, and interpersonal treatment. These findings substantiate that of Chandler et al. (2019), Seifi et al. (2020), Bamba et al. (2021), and Cor et al. (2021), who reported applicants’ satisfaction with VI concerning financial issues and travel distance. Travel and residency costs are among the main concerns for people involved in on-site interviewing (Seifi et al., 2020), associated with obligating to pay and leading to inefficiency (Molina et al., 2020). Some virtual world experts conceive this crisis period as an opportunity for students to experience more time-saving and less stressful recruitment processes (Sullivan et al., 2020). As the pandemic crisis will continue, consequent candidate recruitment programs should think innovatively and deem the situation as “an opportune time” (Tseng, 2020).

The least satisfying features of the VI were perceived behavioral control, the ability to communicate information/opportunity to perform, and procedural justice. A possible explanation for not feeling enough behavior control on the applicants’ part may be related to professors, as the meeting hosts are responsible for giving the interviewees audio and video accessibility. When applicants feel such a lack of control in a structured VI context, they react negatively against the experience (Deiorio et al., 2019). Generally, interviewees are evaluated on their communicative abilities, and VI is disadvantageous in this regard as applicants’ personalities cannot be shown in such communication (Hudak et al., 2019). Furthermore, the lack of feedback in VI invokes the applicants’ impression that they have not been given enough chance to perform (Langer et al., 2017). According to social presence theory (Short et al., 1976), when parties feel the communication partners’ presence, they favor the conversation more, which also impacts their perceptions and communication quality. Likewise, partner presence leads to richer social cues, contributing to better social bandwidth (Potosky, 2008). This lack of opportunity to achieve explains our study participants’ adverse reactions against VI’s procedural justice. However, the VI procedure was considered fair by most participants in Deiorio et al. (2019). To minimize these adverse reactions and present their best version, applicants should optimize their surroundings, check their audiovisual quality, and pay close attention to the overall etiquettes (Sarac et al., 2020).

Among all survey subscales, privacy concerns was the one about which most participants were uncertain as 58% had no idea about it. As suggested by the media attributes framework, privacy concerns are closely connected to surveillance issues. Interview sessions may be recorded and stored clandestinely, and the applicants may be unaware of them (Melchers et al., 2021). This uncertainty may affect their reactions and performance and the impression that VI does not respect their privacy (Balcerak and Woźniak, 2021). Gender differences did not significantly impact the applicants’ perceptions, as was also the case in Baker et al. (2020) and Seifi et al. (2020). But Shreffler et al. (2021) found that male and female applicants differed regarding their responses to some survey statements. At the time of this study, 75% of our participants did not have any prior experience with VI. The results revealed that VI did not impact the applicants’ perceptions which is in line with Basch et al. (2020a) but contradicts McColl and Michelotti (2019).

The three major categories of VI benefits had to do with financial, cognitive, and personal issues. Pre- and during-pandemic research gives some accounts of the same benefits as admitted by participants. Less stress and frustration, more efficiency in time and travel, and the chance to take part in several interviews are only some of these benefits (Melendez et al., 2012; Watson et al., 2017; Vining et al., 2020). Financial and temporal savings are welcomed by both interviewers and interviewees, especially for the latter. They can afford more interview sessions and do not have to decline some of them; moreover, being safe from unexpected hazardous travels is just another gift for them (Joshi et al., 2020). Some of the Ph.D. applicants had jobs that might cause them some scheduling problems for the interview sessions. VI alleviated these problems by offering flexible and convenient scheduling (Bhardwaj et al., 2021).

The main drawbacks associated with VI were technical, communicative, and personal challenges experienced by the applicants. Less chance for self-representation was a drawback of VI, as evident in the qualitative data. This finding contradicts those of Healy and Bedair (2017), Chandler et al. (2019), Grova et al. (2020), Vining et al. (2020), and Hill et al. (2021b), in which applicants admitted the opportunity to represent themselves accurately *via* videoconference interviewing. Ph.D. applicants had some complaints about not receiving enough feedback from the professors. According to Majumder et al. (2020), some feedback provision features characteristic of face-to-face interviews, such as non-verbal cues, cannot be easily replicated in VI. One of the most important non-verbal cues is eye contact which plays a significant role in social judgment (Leung et al., 2021). In VI, applicants report “suboptimal eye contact” as well as difficulty in conversations (Davis et al., 2021). Limited or omitted eye contact is an inseparable feature of VI (Hudak et al., 2019). Although Potosky (2008) admitted that traditional interviews and VI are both interactive, they provide non-equivalent social bandwidth since partners’ complete and detailed behavior cannot be seen *via* a computer screen (Balcerak and Woźniak, 2021). In VI, interaction is further limited by lag times (Wegge, 2006) and less opportunity to perform non-verbal behavior (Toldi, 2011; Blacksmith et al., 2016). This limited opportunity is closely related to the transparency attribute, as communication is mediated by webcams and microphones (Basch et al., 2020a). Another communicative aspect being criticized by our study participants was having no access to all professors’ videos, which contrasts with Majumder et al. (2020) who reported that ease of interaction with all interview members was a positive aspect mentioned by applicants. Virtual platforms generally do not allow the personal interaction typical in face-to-face interviews (Schlitzkus et al., 2013; McKinley et al., 2020). The latter provides the highest transparency level (Melchers et al., 2021) as a crucial component of achieving mutual, two-way information transfer. Communication can also be inhibited by the concern that individuals other than professors will hear it at the moment or by the concern that the interview session will be recorded for subsequent evaluation. Thus, as one of the media attributes, surveillance issues can lead to modifications in the assessment process *via* VI and the applicants’ perceptions toward it (Potosky, 2008).

On the contrary, Davis et al.’s (2020) participants complained that distinguishing the speaker in a single conference room was difficult and did not allow focused conversations. As mentioned previously, VI can be a double-edged sword (Clary, 2021) as it can yield both personal challenges and benefits. So, while VI decreases some face-to-face interview problems, it may not yield the same goals achieved *via* on-site interviewing (Wolff and Burrows, 2020).

Most of these communicative problems cited by applicants were somehow associated with the inherent technical challenges, such as webcam and audio quality which result in poor mutual interaction (Bohannon et al., 2013; Leung et al., 2021), and “dynamics of group interactions” can be threatened by connectivity interruptions as well (Foong, 2020). Among the technical challenges, video and voice interruptions were more often highlighted by the applicants, which were the same problems for participants in Shah et al. (2012) and Edje et al. (2013). As media attributes and communication channels in VI, audio and video impairment result in negative justice perceptions as well as limited social bandwidth (Basch et al., 2020a). Applicants and faculty should also bear in mind that “glitches” may occur unexpectedly (Feld and Shah, 2021). As technical problems cannot be expected, it is better to have a kind of backup program to save time and allow all applicants to contribute (Hill et al., 2021a). The applicants themselves should also think of the same issues and test their technology quality in advance (Jones and Abdelfattah, 2020; McKinley et al., 2020). Besides, not all applicants will be ready to accept VI as they may not have access to good-quality internet and technological support like a webcam (Pourmand et al., 2018). Unfortunately, VI settings can bring about unwanted bias due to differences in applicants’ technical capabilities (Patel et al., 2020). This “implicit bias” should be mitigated by training the applicants as well as the faculty to recognize the bias and necessary techniques for performing in VI (Nwora et al., 2021).

Applicants were also dissatisfied with the non-personal and non-academic atmosphere they experienced during the VI. In this regard, on-site interviews are superior to VIs for recruiting and selecting purposes as they allow a mutual interaction between applicants and program members as well as feeling for the program and its location (Shappell and Schnapp, 2019). As the applicants may be admitted to several universities, they have to make decisions and this decision making is significantly impacted by visiting the interview campus, which gives a fuller understanding of the applicants’ potential “future home” (Bernstein et al., 2020).

Considering all the positive and negative points highlighted by the Ph.D. applicants, they offered two ways to assist for a more efficient future VI. Holding webinars and sharing tutorial videos were deemed to be helpful in this regard. Providing pre-interview preparation and distributing pre-interview materials for both applicants and faculty are among important strategies to consider when assessing and recruiting applicants online (Chou et al., 2020; Hill et al., 2021b), such as holding mock interviews and designing methods to familiarize the applicants with the procedure such as videos or podcasts (Vining et al., 2020).

## Conclusion

This study offered empirical findings suggesting that VIs can be used successfully for future educational entrance programs. The interview findings shed further light on the initial quantitative results offering that VI can both contribute to and hinder the flow of the interview, which can be optimized by preparing applicants in advance for such experience. Several limitations were inherent in this study; for example, more applicants from different fields of study could be surveyed to reach more generalizable data with a more representative sample size. Other researchers are recommended to investigate the faculty’s reflections on using VIs for Ph.D. entrance. Another interesting line of future inquiry may be exploring major and digital literacy’s effects on applicants’ reflections. Such studies may be well accompanied by investigating other individual differences like technophobia, anxiety, and personality types. Although the participants were assured that their responses and comments would have no impact on the interview results, there is the potentiality that they may be biased in favor of the VI. About the implications of this study in recruitment programs, it is recommended that administrators of Ph.D. programs raise applicants’ awareness of VIs procedure and enhance their applicability for the entrance interviews.

## Data Availability Statement

The raw data supporting the conclusions of this article will be made available by the authors, without undue reservation.

## Author Contributions

Both authors listed have made a substantial, direct, and intellectual contribution to the work, and approved it for publication.

## Conflict of Interest

The authors declare that the research was conducted in the absence of any commercial or financial relationships that could be construed as a potential conflict of interest.

## Publisher’s Note

All claims expressed in this article are solely those of the authors and do not necessarily represent those of their affiliated organizations, or those of the publisher, the editors and the reviewers. Any product that may be evaluated in this article, or claim that may be made by its manufacturer, is not guaranteed or endorsed by the publisher.

## Appendix A: The English version of the online survey

**Table:**

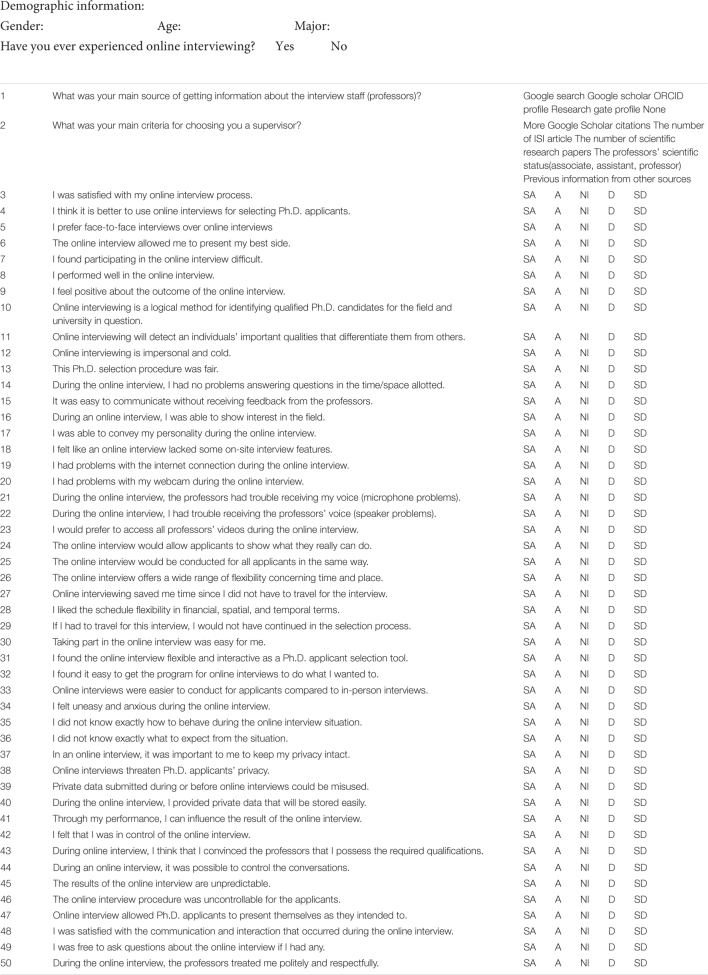

## Appendix B: The guiding interview questions

(1) What benefits did you perceive in online interviews that were absent in on-site interviews?
(2) What were the challenges associated with online interviews that were not characteristic of the on-site interviews?
(3) How can the universities and departments best portray and explain online interview procedures?
(4) How can the Ph.D. applicant best learn about the online interview and its environment?

## References

[B1] Al Saiegh F. Ghosh R. Stefanelli A. Khanna O. Hattar-Medina E. Hoffman M. (2020). Virtual residency training interviews in the age of COVID-19 and beyond. *World Neurosurg.* 143 641–643. 10.1016/j.wneu.2020.08.144 33036950 PMC7538080

[B2] Alhojailan A. I. (2020). The effect of interviewers’ genders on the quantity and quality of their interviewees’ output: a comparative inquiry among Saudi students. *Int. J. English Lang. Educ.* 8 137–150. 10.5296/ijele.v8i2.17377

[B3] Arthur W. Keiser N. L. Doverspike D. (2018). An information-processing-based conceptual framework of the effects of unproctored internet-based testing devices on scores on employment-related assessments and tests. *Hum. Perform.* 31 1–32. 10.1080/08959285.2017.1403441

[B4] Baker D. A. Burns D. M. Reynolds Kueny C. (2020). Just sit back and watch: large disparities between video and face-to-face interview observers in applicant ratings. *Int. J. Hum. Comp. Interact.* 36 1968–1979. 10.1080/10447318.2020.1805874

[B5] Balcerak A. Woźniak J. (2021). The synchronous video interviews in personnel selection processes. *Eur. Res. Stud.* 24 3–13.

[B6] Bamba R. Bhagat N. Tran P. C. Westrick E. Hassanein A. H. Wooden W. A. (2021). Virtual interviews for the independent plastic surgery match: a modern convenience or a modern misrepresentation? *J. Surg. Educ.* 78 612–621. 10.1016/j.jsurg.2020.07.038 32958417 PMC7500901

[B7] Barbour M. E. Sandy J. R. (2014). Multiple mini interviews for selection of dental students: influence of gender and starting station. *J. Dent. Educ.* 78 589–596. 10.1002/j.0022-0337.2014.78.4.tb05710.x 24706689

[B8] Barry B. Fulmer I. S. (2004). The medium and the message: the adaptive use of communication media in dyadic influence. *Acad. Manage. Rev.* 29 272–292. 10.5465/amr.2004.12736093

[B9] Basch J. M. Melchers K. G. (2019). Fair and flexible?! Explanations can improve applicant reactions toward asynchronous video interviews. *Pers. Assess. Decis.* 5 1–11. 10.25035/pad.2019.03.002

[B10] Basch J. M. Melchers K. G. Kegelmann J. Lieb L. (2020a). Smile for the camera! The role of social presence and impression management in perceptions of technology-mediated interviews. *J. Manag. Psychol.* 35 285–299. 10.1108/JMP-09-2018-039

[B11] Basch J. M. Melchers K. G. Kurz A. Krieger M. Miller L. (2020b). It takes more than a good camera: which factors contribute to differences between face-to-face interviews and videoconference interviews regarding performance ratings and interviewee perceptions? *J. Bus. Psychol.* 36 921–940. 10.1007/s10869-020-09714-3 32929301 PMC7482058

[B12] Bernstein S. A. Gu A. Chretien K. C. Gold J. A. (2020). Graduate medical education virtual interviews and recruitment in the era of COVID-19. *J. Graduate Med. Educ.* 12 557–560. 10.4300/JGME-D-20-00541.1 33149823 PMC7594783

[B13] Bhardwaj P. Kleiber G. M. Baker S. B. Fan K. L. (2021). Applying to residency in the COVID-19 era: virtual interview tips for success. *Plastic Reconstruct. Surg. Glob. Open* 9 1–2. 10.1097/GOX.0000000000003389 33564599 PMC7862738

[B14] Blacksmith N. Willford J. C. Behrend T. S. (2016). Technology in the employment interview: a meta-analysis and future research agenda. *Pers. Assess. Decis.* 2 12–20. 10.25035/pad.2016.002

[B15] Bohannon L. S. Herbert A. M. Pelz J. B. Rantanen E. M. (2013). Eye contact and video-mediated communication: a review. *Displays* 34 177–185. 10.1016/j.displa.2012.10.009

[B16] Byrne D. (1961). Interpersonal attraction as a function of AfViation need and attitude similarity. *Hum. Relat.* 14 283–289. 10.1177/001872676101400305

[B17] Chandler N. M. Litz C. N. Chang H. L. Danielson P. D. (2019). Efficacy of videoconference interviews in the pediatric surgery match. *J. Surg. Educ.* 76 420–426. 10.1016/j.jsurg.2018.08.010 30219521

[B18] Chapman D. S. Uggerslev K. L. Webster J. (2003). Applicant reactions to face-to-face and technology-mediated interviews: a field investigation. *J. Appl. Psychol.* 88 944–953. 10.1037/0021-9010.88.5.944 14516254

[B19] Chou D. W. Pletcher S. D. Bruss D. Sung C. K. Diaz R. C. Liang J. (2020). Otolaryngology residency interviews in a socially distanced world: strategies to recruit and assess applicants. *Otolaryngol. Head Neck Surg.* 164 903–908. 10.1177/0194599820957961 32870721

[B20] Clary K. L. (2021). Considering a new platform for academic campus interviews: entering the virtual world. *Qual. Soc. Work* 20 610–617. 10.1177/1473325020981072

[B21] Cor K. Snghera R. Brocks D. R. (2021). Online interviews for the selection of applicants for admission into an entry to practice program in pharmacy: relationship to performance and student perspectives. *Curr. Pharm. Teach. Learn.* 13 616–622. 10.1016/j.cptl.2021.01.033 33867055

[B22] Creswell J. W. Plano Clark V. L. Gutmann M. Hanson W. (2003). “Advanced mixed methods research designs,” in *Handbook on Mixed Methods in the Behavioral and Social Sciences*, eds Tashakkori A. Teddlie C. (Thousand Oaks, CA: Sage Publications), 209–240.

[B23] Davis M. E. Jafari A. Crawford K. MacDonald B. V. Watson D. (2021). Novel implementation of virtual interviews for otolaryngology resident selection: reflections relevant to the COVID-19 era. *OTO Open* 5 1–4. 10.1177/2473974X20988234 33598597 PMC7863174

[B24] Davis M. G. Haas M. R. Gottlieb M. House J. B. Huang R. D. Hopson L. R. (2020). Zooming in versus flying out: virtual residency interviews in the era of COVID-19. *AEM Educ. Train.* 4 443–446. 10.1002/aet2.10486 33150292 PMC7592818

[B25] Day R. W. Taylor B. M. Bednarski B. K. Tzeng C. W. D. Gershenwald J. E. Lee J. E. (2020). Virtual interviews for surgical training program applicants during COVID-19: lessons learned and recommendations. *Ann. Surg.* 272 e144–e147. 10.1097/SLA.0000000000004064 32675519 PMC7268876

[B26] Deiorio N. M. Jarou Z. J. Alker A. Bird S. B. Druck J. Gallahue F. E. (2019). Applicant reactions to the AAMC standardized video interview during the 2018 application cycle. *Acad. Med.* 94 1498–1505. 10.1097/ACM.0000000000002842 31219811

[B27] Dornyei Z. (2007). *Research Methods in Applied Linguistics: Quantitative, Qualitative, and Mixed Methodologies.* Oxford: Oxford University Press.

[B28] Edje L. Miller C. Kiefer J. Oram D. (2013). Using Skype as an alternative for residency selection interviews. *J. Graduate Med. Educ.* 5 503–505. 10.4300/JGME-D-12-00152.1 24404318 PMC3771184

[B29] Feld L. D. Shah N. L. (2021). Connecting during the virtual interview process: lessons from experience. *Digestive Dis. Sci.* 66 1–2. 10.1007/s10620-020-06744-y 33433799 PMC7801879

[B30] Foong C. C. (2020). Preliminary measures in the COVID-19 pandemic: a trial for futuristic medical education. *J. Res. Med. Dent. Sci.* 8 77–78.

[B31] Grova M. M. Donohue S. J. Meyers M. O. Kim H. J. Ollila D. W. (2020). Direct comparison of in-person versus virtual interviews for complex general surgical oncology fellowship in the COVID-19 era. *Ann. Surg. Oncol.* 28 1908–1915. 10.1245/s10434-020-09398-2 33244739 PMC7690846

[B32] Hammoud M. M. Standiford T. Carmody J. B. (2020). Potential implications of COVID-19 for the 2020-2021 residency application cycle. *JAMA* 324 29–30. 10.1001/jama.2020.8911 32492097

[B33] Hannon B. (2012). Test anxiety and performance-avoidance goals explain gender differences in SAT-V, SAT-M, and overall SAT scores. *Pers. Individ. Differ.* 53 816–820. 10.1016/j.paid.2012.06.003 23997382 PMC3756515

[B34] Harley J. M. Lou N. M. Liu Y. Cutumisu M. Daniels L. M. Leighton J. P. (2020). University students’ negative emotions in a computer-based examination: the roles of trait test-emotion, prior test-taking methods and gender. *Assessment Eval. Higher Educ.* 46 1–17. 10.1080/02602938.2020.1836123

[B35] Healy W. L. Bedair H. (2017). Videoconference interviews for an adult reconstruction fellowship: lessons learned. *JBJS* 99:e114. 10.2106/JBJS.17.00322 29088046

[B36] Hill M. V. Ross E. A. Crawford D. Lai L. Turaga K. Grubbs E. G. (2021b). Program and candidate experience with virtual interviews for the 2020 Complex General Surgical Oncology interview season during the COVID pandemic. *Am. J. Surg.* 222 99–103. 10.1016/j.amjsurg.2020.11.007 33189309

[B37] Hill M. V. Bleicher R. J. Farma J. M. (2021a). A how-to guide: virtual interviews in the era of social distancing. *J. Surg. Educ.* 78 321–323. 10.1016/j.jsurg.2020.07.016 32741692 PMC7391954

[B38] Hudak K. Kile A. Grodziak E. Keptner E. (2019). Advancing student interview skills: incorporating virtual interview technology into the basic communication course. *International J. Scholarship Teach. Learn.* 13 1–10. 10.20429/ijsotl.2019.130103

[B39] Huffcutt A. I. Van Iddekinge C. H. Roth P. L. (2011). Understanding applicant behavior in employment interviews: a theoretical model of interviewee performance. *Hum. Resour. Manage. Rev.* 21 353–367. 10.1016/j.hrmr.2011.05.003

[B40] Jones R. E. Abdelfattah K. R. (2020). Virtual interviews in the era of COVID-19: A primer for applicants. *J. Surg. Educ.* 77 733–734. 10.1016/j.jsurg.2020.03.020 32278546 PMC7142702

[B41] Joshi A. Bloom D. A. Spencer A. Gaetke-Udager K. Cohan R. H. (2020). Video interviewing: a review and recommendations for implementation in the era of COVID-19 and beyond. *Acad. Radiol.* 27 1316–1322. 10.1016/j.acra.2020.05.020 32563558 PMC7833741

[B42] Kamboj A. K. Raffals L. E. Martin J. A. Chandrasekhara V. (2021). Virtual interviews during the COVID-19 pandemic: a survey of advanced endoscopy fellowship applicants and programs. *Tech. Innov. Gastrointest. Endosc.* 23 159–168. 10.1016/j.tige.2021.02.001 34697608 PMC8528488

[B43] Kenwell O. K. Mahoney B. P. Rosenblatt M. A. (2021). A response to the challenges of virtual interviews and recruitment in the era of COVID-19. *J. Graduate Med. Educ.* 13 134–136. 10.4300/JGME-D-20-01288.1 33680316 PMC7901611

[B44] Langer M. König C. J. Krause K. (2017). Examining digital interviews for personnel selection: applicant reactions and interviewer ratings. *Int. J. Selection Assessment* 25 371–382. 10.1111/ijsa.12191

[B45] Leduc J. M. Rioux R. Gagnon R. Bourdy C. Dennis A. (2017). Impact of sociodemographic characteristics of applicants in multiple mini-interviews. *Med. Teacher* 39 285–294. 10.1080/0142159X.2017.1270431 28024439

[B46] Leung C. Malone M. Way D. P. Barrie M. G. Kman N. E. San Miguel C. (2021). Preparing students for residency interviews in the age of COVID: lessons learned from a standardized video interview preparation program. *AEM Educ. Train*. 10583 1–9. 10.1002/aet2.10583 33821226 PMC8013192

[B47] Lewit R. Gosain A. (2021). Virtual interviews may fall short for pediatric surgery fellowships: lessons learned from COVID-19/SARS-CoV-2. *J. Surg. Res.* 259 326–331. 10.1016/j.jss.2020.09.029 33127064 PMC7546197

[B48] Lukacik E. R. Bourdage J. S. Roulin N. (2020). Into the void: a conceptual model and research agenda for the design and use of asynchronous video interviews. *Hum. Resour. Manage. Rev.* 10.1016/j.hrmr.2020.100789 [Epub ahead of print].

[B49] Majumder A. Eckhouse S. R. Brunt L. M. Awad M. M. Dimou F. M. Eagon J. C. (2020). Initial experience with a virtual platform for Advanced Gastrointestinal Minimally Invasive Surgery Fellowship interviews. *J. Am. Coll. Surg.* 231 670–678. 10.1016/j.jamcollsurg.2020.08.768 32950602 PMC7497742

[B50] McColl R. Michelotti M. (2019). Sorry, could you repeat the question? Exploring video-interview recruitment practice in HRM. *Hum. Resour. Manage. J.* 29 637–656. 10.1111/1748-8583.12249

[B51] McKinley S. K. Fong Z. V. Udelsman B. Rickert C. G. (2020). Successful virtual interviews: perspectives from recent surgical fellowship applicants and advice for both applicants and programs. *Ann. Surg.* 272 e192–e196. 10.1097/SLA.0000000000004172 32649461

[B52] Melchers K. G. Petrig A. Basch J. M. Sauer J. (2021). A comparison of conventional and technology-mediated selection interviews with regard to interviewees’ performance, perceptions, strain, and anxiety. *Front. Psychol.* 11:603632. 10.3389/fpsyg.2020.603632 33510679 PMC7835329

[B53] Melendez M. M. Dobryansky M. Alizadeh K. (2012). Live online video interviews dramatically improve the plastic surgery residency application process. *Plastic Reconstructive Surg.* 130 240e–241e. 10.1097/PRS.0b013e3182550411 22743950

[B54] Molina G. Mehtsun W. T. Qadan M. Hause K. C. Raut C. P. Fairweather M. (2020). Virtual interviews for the complex general surgical oncology fellowship: the dana-farber/partners experience. *Ann. Surg. Oncol.* 27 3103–3106. 10.1245/s10434-020-08778-y 32613367 PMC7328646

[B55] Nesdoly N. Tulk C. Mantler J. (2020). The effects of perceived professor competence, warmth and gender on students’ likelihood to register for a course. *Assessment Eval. Higher Educ.* 45 666–679. 10.1080/02602938.2019.1689381

[B56] Nikolaou I. (2021). What is the role of technology in recruitment and selection? *Spanish J. Psychol.* 24 1–6. 10.1017/SJP.2021.6 33536110

[B57] Nwora C. Allred D. B. Verduzco-Gutierrez M. (2021). Mitigating bias in virtual interviews for applicants who are underrepresented in medicine. *J. Natl. Med. Asso.* 113 74–76. 10.1016/j.jnma.2020.07.011 32768242 PMC7402105

[B58] Patel T. Y. Bedi H. S. Deitte L. A. Lewis P. J. Marx M. V. Jordan S. G. (2020). Brave new world: challenges and opportunities in the COVID-19 virtual interview season. *Acad. Radiol.* 27 1456–1460. 10.1016/j.acra.2020.07.001 32948443 PMC7362778

[B59] Pogrebtsova E. Luta D. Hausdorf P. A. (2020). Selection of gender-incongruent applicants: no gender bias with structured interviews. *Int. J. Selection Assessment* 28 117–121. 10.1111/ijsa.12270

[B60] Potosky D. (2008). A conceptual framework for the role of the administration medium in the personnel assessment process. *Acad. Manage. Rev.* 33 629–648. 10.5465/amr.2008.32465704

[B61] Pourmand A. Lee H. Fair M. Maloney K. Caggiula A. (2018). Feasibility and usability of tele-interview for medical residency interview. *Western J. Emerg. Med.* 19 80–86. 10.5811/westjem.2017.11.35167 29383060 PMC5785206

[B62] Riazi A. M. (2016). *The Routledge Encyclopedia of Research Methods in Applied Linguistics.* Milton: Routledge.

[B63] Robinson K. A. Shin B. Gangadharan S. P. (2020). A comparison between in-person and virtual fellowship interviews during the COVID-19 pandemic. *J. Surg. Educ.* 78 1175–1181. 10.1016/j.jsurg.2020.11.006 33250429 PMC7678431

[B64] Robles F. Sears G. J. Zhang H. Wiesner W. H. Hackett R. D. Yuan Y. (2013). A comparative assessment of videoconference and face-to-face employment interviews. *Manage. Decis.* 51 1733–1752. 10.1108/MD-09-2012-0642

[B65] Sarac B. A. Calamari K. Janis J. (2020). Virtual residency interviews: optimization for applicants. *Cureus* 12 1–3. 10.7759/cureus.11170 33251076 PMC7688057

[B66] Schlitzkus L. L. Schenarts P. J. Schenarts K. D. (2013). It was the night before the interview: perceptions of resident applicants about the preinterview reception. *J. Surg. Educ.* 70 750–757. 10.1016/j.jsurg.2013.05.008 24209651

[B67] Seifi A. Mirahmadizadeh A. Eslami V. (2020). Perception of medical students and residents about virtual interviews for residency applications in the United States. *PLoS One* 15:e0238239. 10.1371/journal.pone.0238239 32866220 PMC7458290

[B68] Shah S. K. Arora S. Skipper B. Kalishman S. Timm T. C. Smith A. Y. (2012). Randomized evaluation of a web based interview process for urology resident selection. *J. Urol.* 187 1380–1384. 10.1016/j.juro.2011.11.108 22341282

[B69] Shappell E. Schnapp B. (2019). The F word: how “fit” threatens the validity of resident recruitment. *J. Graduate Med. Educ.* 11 635–636. 10.4300/JGME-D-19-00400.1 31871561 PMC6919185

[B70] Short J. Williams E. Christie B. (1976). *The Social Psychology of Telecommunications.* Hoboken, NJ: Wiley.

[B71] Shreffler J. Platt M. Huecker M. (2021). Planning virtual residency interviews as a result of COVID-19: insight from residency applicants and physicians conducting interviews. *Postgraduate Med. J.* [Epub ahead of print]. 10.1136/postgradmedj-2020-139182 33504613

[B72] Spronken-Smith R. Cameron C. Quigg R. (2018). Factors contributing to high PhD completion rates: a case study in a research-intensive university in New Zealand. *Assessment Eval. Higher Educ.* 43 94–109. 10.1080/02602938.2017.1298717

[B73] Strielkowski W. (2020). COVID-19 pandemic and the digital revolution in academia and higher education. *Preprints* 10.20944/preprints202004.0290.v1 32283112

[B74] Sullivan G. M. Deiorio N. M. Yarris L. M. (2020). Teaching, interviewing, and recruitment in the time of COVID-19. *J. Graduate Med. Educ.* 12 523–524. 10.4300/JGME-D-20-01004.1 33149814 PMC7594775

[B75] Toldi N. L. (2010). *Job Applicant Reactions to the Use of Video Interviewing as a Selection Tool*. Unpublished master’s thesis. Pennsylvania: The Pennsylvania State University.

[B76] Toldi N. L. (2011). Job applicants favor video interviewing in the candidate-selection process. *Employment Relations Today* 38 19–27. 10.1002/ert.20351

[B77] Tseng J. (2020). How has COVID-19 affected the costs of the surgical fellowship interview process? *J. Surg. Educ.* 77 999–1004. 10.1016/j.jsurg.2020.05.018 32507697 PMC7237896

[B78] Vining C. C. Eng O. S. Hogg M. E. Schuitevoerder D. Silverman R. S. Yao K. A. (2020). Virtual surgical fellowship recruitment during COVID-19 and its implications for resident/fellow recruitment in the future. *Ann. Surg. Oncol.* 27(Suppl. 3) s911–s915. 10.1245/s10434-020-08623-2 32424589 PMC7233675

[B79] Watson S. L. Hollis R. H. Oladeji L. Xu S. Porterfield J. R. Ponce B. A. (2017). The burden of the fellowship interview process on general surgery residents and programs. *J. Surg. Educ.* 74 167–172. 10.1016/j.jsurg.2016.06.008 27425434

[B80] Wegge J. (2006). Communication via videoconference: emotional and cognitive consequences of affective personality dispositions, seeing one’s own picture, and disturbing events. *Hum. Comp. Interact.* 21 273–318. 10.1207/s15327051hci2103_1

[B81] Wolff M. Burrows H. (2020). Planning for virtual interviews: residency recruitment during a pandemic. *Acad. Pediatr.* 21 24–31. 10.1016/j.acap.2020.10.006 33068812 PMC7558234

[B82] Wong O. (2020). *How Asynchronous Video Interview Design Affects Applicant Outcomes: Interview Performance, Impression Management, Anxiety, and Perceived Fairness*. Unpublished master’s thesis. Halifax: Saint Mary’s University.

